# Male partners of young women in Uganda: Understanding their relationships and use of HIV testing

**DOI:** 10.1371/journal.pone.0200920

**Published:** 2018-08-10

**Authors:** Ann Gottert, Julie Pulerwitz, Godfrey Siu, Anne Katahoire, Jerry Okal, Florence Ayebare, Nrupa Jani, Pamela Keilig, Sanyukta Mathur

**Affiliations:** 1 Population Council, HIV and AIDS Program, Washington D.C., United States of America; 2 Makerere University Child Health and Development Centre, Kampala, Uganda; 3 Population Council, HIV and AIDS Program, Nairobi, Kenya; UNAIDS, UNITED STATES

## Abstract

**Background:**

Substantial concern exists about the high risk of sexually transmitted HIV to adolescent girls and young women (AGYW, ages 15–24) in Eastern and Southern Africa. Yet limited research has been conducted with AGYW’s male sexual partners regarding their perspectives on relationships and strategies for mitigating HIV risk. We sought to fill this gap in order to inform the DREAMS Partnership and similar HIV prevention programs in Uganda.

**Methods:**

We conducted 94 in-depth interviews, from April-June 2017, with male partners of AGYW in three districts: Gulu, Mukono, and Sembabule. Men were recruited at community venues identified as potential transmission areas, and via female partners enrolled in DREAMS. Analyses focused on men’s current and recent partnerships and HIV service use.

**Results:**

Most respondents (80%) were married and 28 years old on average. Men saw partner concurrency as pervasive, and half described their own current multiple partners. Having married in their early 20s, over time most men continued to seek out AGYW as new partners, regardless of their own age. Relationships were highly fluid, with casual short-term partnerships becoming more formalized, and more formalized partnerships characterized by periods of separation and outside partnerships. Nearly all men reported recent HIV testing and described testing at distinct relationship points (e.g., when deciding to continue a relationship/get married, or when reuniting with a partner after a separation). Testing often stemmed from distrust of partner behavior, and an HIV-negative status served to validate respondents’ current relationship practices.

**Conclusions:**

Across the three regions in Uganda, findings with partners of AGYW confirm earlier reports in Uganda of multiple concurrent partnerships, and demonstrate substantial HIV testing. Yet they also unearth the degree to which these partnerships are fluid (switching between casual and/or more long-term partnerships), which complicates potential HIV prevention strategies. Context-specific findings around these partnerships and risk are critical to further tailor HIV prevention programs.

## Introduction

Eastern and Southern Africa, with only 6% of the world’s population, is home to half of the world’s people living with HIV, and contributed 46% of all new HIV infections in 2015 [1]. While substantial progress has been made towards reaching UNAIDS-led goals around treatment and mortality decline, rates of new infections among adults remain static across the region [1, 2]. Uganda, with about 1.3 million adults aged 15 to 49 living with HIV in the country and a prevalence of 7.3% [3], has recently seen some increases in both HIV prevalence and incidence [3, 4].

Adolescent girls and young women (AGYW) are particularly vulnerable to HIV in Uganda, with a prevalence of 3.7% among young women ages 15–24 versus 2.3% among men the same age [3]. There is extensive evidence that multiple partnerships are linked to HIV risk for men and women, and that characteristics of age-disparate partnerships increase risk for AGYW [5–8]. Consequently, there has been recent increased attention on male partners within HIV prevention efforts for AGYW [9, 10]. A prominent example is the PEPFAR-supported DREAMS Partnership [11], which aims to decrease HIV incidence among AGYW across ten countries, including Uganda, by providing a package of evidence-based interventions, and intervening not only with AGYW but with their sexual partners, parents and communities Regarding men, DREAMS is particularly focused on characterizing male partners of AGYW and engaging them in HIV services including HIV testing, voluntary medical male circumcision (VMMC), and antiretroviral treatment (ART) [11]. Across the region, men tend to test for HIV and enter ART programs at a more advanced clinical stage than women and, as a consequence, have substantially higher morbidity and mortality [1, 12].

However, in Uganda and across the region, there remains a knowledge gap around men’s perspectives on partnerships with young women and how they understand and mitigate HIV risk within those partnerships. Most prior research to understand power dynamics and HIV risk within relationships, and particularly young women’s relationships, has been conducted with women rather than men [9]. Understanding men’s perspectives on such issues may be key to designing more nuanced messages and strategies to constructively engage them in HIV prevention [7, 13, 14]. In particular, in order to build an effective HIV prevention response in each country and locality, it is imperative to orient programming to individuals’ patterns of relationships and health service needs over time [10]. Such patterns can be difficult to capture and disentangle via quantitative surveys, which instead often capture a more ‘cross-sectional’ or partner-specific view—for example, the percent who had multiple sexual partners in a certain timeframe, or percent who tested for HIV with their most recent partner. In contrast, qualitative research is ideally positioned to capture patterns and motivations since participants have the opportunity to explain their experiences over time in their own words [15].

The purpose of this study was therefore to hear from men in three geographically and culturally diverse Ugandan districts where DREAMS is being implemented—Gulu, Mukono and Sembabule—about their relationships, particularly with young women, and how and when they considered and used HIV services within these relationships. Findings are intended to inform the development and refinement of effective HIV prevention strategies in Uganda, including the DREAMS Partnership.

## Methods

### Study sites

This qualitative study took place in three Ugandan districts: Gulu, Mukono, and Sembabule. Each of these districts is participating in the DREAMS program. Within each district, interviews were conducted in one rural and one urban site. These districts and study sites within districts were identified through consultation with local stakeholders. By locating the study across diverse geographic regions and urban/rural areas we sought to explore the diversity of relationships between the at-risk men and young women that programming is seeking to serve. Gulu is a post-conflict area in the north of the country where farming is the main source of income, although its urban areas are fast commercializing. Mukono, just east of the capital Kampala, is relatively urban, with a sizable population engaged in fishing activities on bordering Lake Victoria. Sembabule is on a major trucking route southwest of Kampala and has both urban and rural areas, with a large pastoral cattle raring community.

### Sample and data collection

Between April and June 2017, we conducted 94 in-depth interviews (IDIs) with male partners of AGYW: 30 in Gulu, 33 in Mukono, and 31 in Sembabule. To capture a diversity of types of relationships between men and AGYW, we sought to recruit men who were in more long-term and stable relationships with AGYW (who we anticipated would more likely have monogamous and more equitable relationships), as well as men in more short-term, casual relationships with AGYW (who we anticipated may have multiple partners and less equitable relationships). To reach men in potentially more stable relationships, we recruited male partners of AGYW enrolled in DREAMS, through DREAMS implementing partner (IP) organizations. To reach men in potentially less stable relationships, we recruited men from community venues identified as potential transmission areas (PTAs) in the community—specifically, places where men and young women are believed to meet casual sexual partners.

For male partners of AGYW enrolled in DREAMS, eligible male partners included those who were voluntarily listed by AGYW DREAMS participants, who DREAMS IPs had consent from AGYW to contact, and who DREAMS IPs had already contacted to offer them HIV services. The DREAMS IP at the site made initial contact with the potential participant, and if they were age 18+ and indicated willingness to learn more about the study, they were then connected with the research assistants.

PTA venues were identified during nine focus groups discussions (three per district) conducted from March-April 2017 with key informants including DREAMS IPs, HIV service providers, and community opinion leaders, and/or by DREAMS IPs assisting with recruitment. Types of venues included bars, lodges, boda boda (motor cycle taxi) stands, construction sites, marketplaces, and quarries, among others. At the venue, an eligibility screening form was administered to participants who expressed interest in participating, to ensure that the men were aged 18+, and had at least 1 female partner age <25 in last year (i.e., were a male partner of AGYW).

All data were collected in the local languages (Luganda and Luo) by six male research assistants (two per district) who had extensive qualitative interviewing experience. Participants were interviewed alone in a private location. Written informed consent was sought and obtained from all participants. Interviews lasted approximately 1–1.5 hours, were guided by a semi-structured interview guide, and were audio recorded. The interview guide explored respondents’ perceptions of men’s relationships with AGYW in their community, their own relationship dynamics, and HIV preventive behaviors across up to their last three partnerships (with a series of questions asked about each partner), and their history of HIV service use. Because only four of the 94 participants reported being HIV-positive, discussions about HIV service use focused primarily on men’s HIV testing practices over time.

### Data analysis

The audio recordings were transcribed and translated into English by the research assistants, and checked by the study coordinator and local principal investigator for completeness and accuracy of translation. We used a spreadsheet to track demographic and other characteristics of interest (e.g., reported HIV testing in the last two years) for each participant. This information was used to group transcripts into sub-groups (e.g. by district) and to generate frequencies of participant characteristics. This was particularly useful given the relatively large sample size for this qualitative study.

Transcripts were coded in Atlas.ti v7 software, and subjected to thematic analysis [16]. The analysis focused on relationship patterns and HIV testing. A code book was developed with topical codes (i.e., based on topics identified in the interview guide) as well as emergent codes (i.e., new topics that emerged during the course of interviewing). A sample of three interviews was coded by three separate study team members, and the coding was compared in order to maximize internal validity. The code book was then finalized by the principal investigators, resulting in 23 main codes.

Study team members then analyzed transcripts using the final code book and prepared summary reports for key codes. Checks to ensure internal validity and consistency in the application of the codes were conducted on a periodic basis by one of the principal investigators. The study team reviewed all summary reports together and came to agreement regarding emerging patterns and themes. Finally, a half-day data interpretation workshop was held with approximately 50 stakeholders in Kampala, Uganda in October 2017. Local and international investigators, programmers, and policy-makers were all in attendance. Feedback from this workshop was incorporated into final data analysis and write-up of results.

### Ethical approval

The study was reviewed and approved by the Population Council IRB as well as the Makerere University School of Public Health Institutional Review Board (IRB), and received final approval from the Uganda National Council for Science and Technology.

## Results

The demographic characteristics of the 94 men interviewed are presented in Table 1. The mean age of the respondents was 28 years, with a range of 19–45 years. Most men (80%) reported being married or cohabiting. About two-thirds had attained at least primary education, and nearly all said that they were currently working. The most common occupations across the districts were boda boda (motorcycle taxi transport); small business, and casual work in building/construction. Additionally, being a fisherman was a common occupation in Mukono, as was being a farmer in Gulu. Over one-third of participants reported being away from home for at least a month in the last year. Nearly half (49%) reported being engaged in multiple concurrent sexual partnerships and 95% reported being tested for HIV in the last two years. Socio-demographic characteristics did not differ markedly between districts, with the exception of certain occupations, as noted above.

**Table 1:** Respondent characteristics (n = 94).

CHARACTERISTIC	n (%)
**District**	
Gulu	30 (32%)
Mukono	33 (35%)
Sembabule	31 (33%)
**Recruitment strategy**	
Recruited at PTA venues	40 (43%)
Recruited via AGYW participating in DREAMS	54 (57%)
**Age—Mean (range)**	28 (19–45)
**Married/cohabiting**	75 (80%)
**Completed at least primary level education**	63 (67%)
**Away from home for at least one month in last year**	39 (41%)
**Currently working (formal or informal employment)**	84 (94%)*
**Most common occupations**	• Boda boda (motorcycle taxi driver)
	• Small business
	• Building/construction
	• Fisherman (Mukono only)
	• Farmer (Gulu only)
**Had multiple concurrent sexual partners in the last two years**	46 (49%)
**Had an HIV test in the last two years**	89 (95%)

*5 responses were missing

Of note, 90% of the men recruited via their AGYW partners in DREAMS were married, versus 63% of men recruited at PTA venues. In addition, fewer of the men recruited via AGYW participating in DREAMS, than those from PTA venues, reported being away from home for at least one month in the last year (38% vs 53%).

Below, we describe the major themes that emerged from data analysis, around: multiple concurrent relationships, men’s sexual relationships, ‘side’ partners, casual transactional partnerships and HIV testing for men. We did not find major differences when comparing themes across the three districts, and therefore present most results reflecting men’s responses across the three districts. In a few notable instances, we highlight differences between men’s responses by district.

### Pervasive multiple concurrent relationships

When asked about the nature of men’s sexual relationships in their community, nearly all of the men described that multiple sexual partnerships are pervasive. Men spoke openly about this practice and often framed its magnitude as a change from the past, using phrases like “these days” and nowadays”.

“These days most men have more than one woman, most men say that you can’t keep eating one type of food all the time. In fact you can’t find a man with only one woman, it isn’t there. Even me, I don’t have only one woman.”

(Age 38, Gulu)

“Nowadays in my community, it is impossible for a man to have one partner especially the men around 25, 30 years…It is very common, although they cannot say it out but they have around 2, 3 partners.”

(Age 40, Sembabule)

About half of the 94 respondents reported having engaged in multiple overlapping relationships in the last two years. A further quarter—while they did not report having multiple concurrent partners—described a lack of interest in maintaining a monogamous relationship and instead expressed interest in ‘side’ partners. Most respondents described side relationships as additional long-term partners, who men often referred to as ‘wives’ and for whom they played the role of the economic provider. Typically, these secondary partners did not live with the respondent or in the same household as his primary partner, and in some cases lived in a different geographic location and only saw the respondent periodically.

"She is not at my home she is in a rental but it's me who pays her rent, it's me who is taking care of her in everything even though she is not at my place she still is like my wife now.”

(Age 35, Sembabule)

Interestingly, men recruited via their AGYW partners enrolled in DREAMS programming were as likely as hot-spot-recruited men to report having multiple concurrent partners, despite the fact that nearly all were married/cohabiting (vs. less than two-thirds of hot-spot-recruited men).

For the quarter of the sample who described maintaining a monogamous relationship and intentionally *not* seeking other partners, cost was noted as the main driving factor in their inability to have another partner. As one man put it, *“I don’t want to have more than one wife because I am not capable of taking care of more than one woman…” (Age 25*, *Mukono)* A number of men described avoiding casual partners entirely, having come to see these relationships as a waste of money.

“Do you know how much I would spend if I chose to have sex with a woman?…For me when I think about wasting money in short time like that, I give it up and stay with my wife.”

(Age 22, Mukono)

### Men’s relationships with AGYW over their life course

Over their life course, and regardless of how old the men were, men described a pattern of establishing relationships with adolescent girls and young women aged between 15–24 years. Most men had married in their early 20s, usually to a woman 3–5 years younger than themselves, and generally perceived of marriage as an important life event and benchmark. In most cases marriage was loosely defined as an ongoing, cohabiting relationship, which may or may not be legal or formalized through payment of bride wealth or by a religious ceremony. Within a few years after marrying, however, many men described taking on one or more side partners. Sometimes this occurred during a period of separation from the first wife due to conflict in that relationship, as described in the second quote below.

“Personally, I have more than three women in this town. I have a wife at home and two side dishes … I would say the majority [of men around here] have more than one wife.”

(Age 38, Sembabule)

“…in case of any small conflict at home between me and my wife, [she] goes back to her home. To me as a man, I may feel like bringing another woman…immediately my former wife hears that I have brought another woman, she may decide to come back to me.”

(Age 24, Gulu)

Separation from a primary partner was often described as being temporary, ranging from a week to several months or longer. Regardless of the length of the separation, men perceived these as opportunities to seek an additional partner or “wife.”

Regardless of the respondent’s own age, side partners generally were adolescent girls or young women when the relationship started. Many men explicitly described seeking out younger women because they were more respectful and caring towards them than women closer to their own age.

“…if you get a woman who is your age mate, somehow these women tend not to be submissive…yet for a young girl, because of the age difference, she will find it very easy to listen to you, she will treat you with respect because she knows you are older than her and perhaps more experienced‥”

(Age 31, Mukono)

### Short-term partners often screened as potential wives

Some men described seeking out a short-term relationship as a trial period for becoming a longer-term partner or wife. In addition to assessing a potential partner’s physical attractiveness, a young woman’s character was also assessed through her manners, respectfulness and industriousness. Men often accomplished this assessment by first asking about the young women and their families, then by interacting with them directly. These relationships were mainly initiated at non-PTA venues, such as at work, school, or church.

“My brother was studying with her in the same school…I asked him what he thinks about her, and he said she is a good girl and her behaviors are not bad. I then told him to invite her to our place one day, which happened…then she went back home. This went on for a long time and the rest is history.”

(Age 27, Gulu)

A number of respondents in Gulu drew specific attention to a local practice of men taking ‘Ogek’ as additional wives. Ogek were described as young women coming out of “failed” marriages and who may already have children. Men perceive these women as good choices for second, third or even fourth wives because they are seen as ‘damaged goods’ and so may demand less of the man financially, and show him more respect.

### Yet, casual transactional relationships were also common

About half of respondents noted that they had short-term casual sexual partners, either instead of or in addition to long-term relationships. These relationships were nearly always transactional in nature and were often initiated at PTA venues within the community like a bar or nightclub. The relationship usually started with the man buying the woman drinks (alcoholic and/or non-alcoholic), a meal, or low cost goods like cell phone credit. Having sex workers as partners was also frequently described in Sembabule and Mukono districts. Men noted that this was a lower cost option compared to taking on another wife or a long-term side partner. The age of sex workers was most often described as early to late 20s, although a number of men in Sembabule highlighted younger groups of sex workers, such as those starting at ages 14 or 15.

“Here a poor man will postpone relationships until he gets the money. Immediately when he (poor man) gets the money, he will visit the market to buy prostitutes instead of ‘marrying’ more than one sexual partner. This is because it is cheaper and sustainable in terms of his financial position.”

*(Age 41, Sembabule)*.

**Fig 1:**
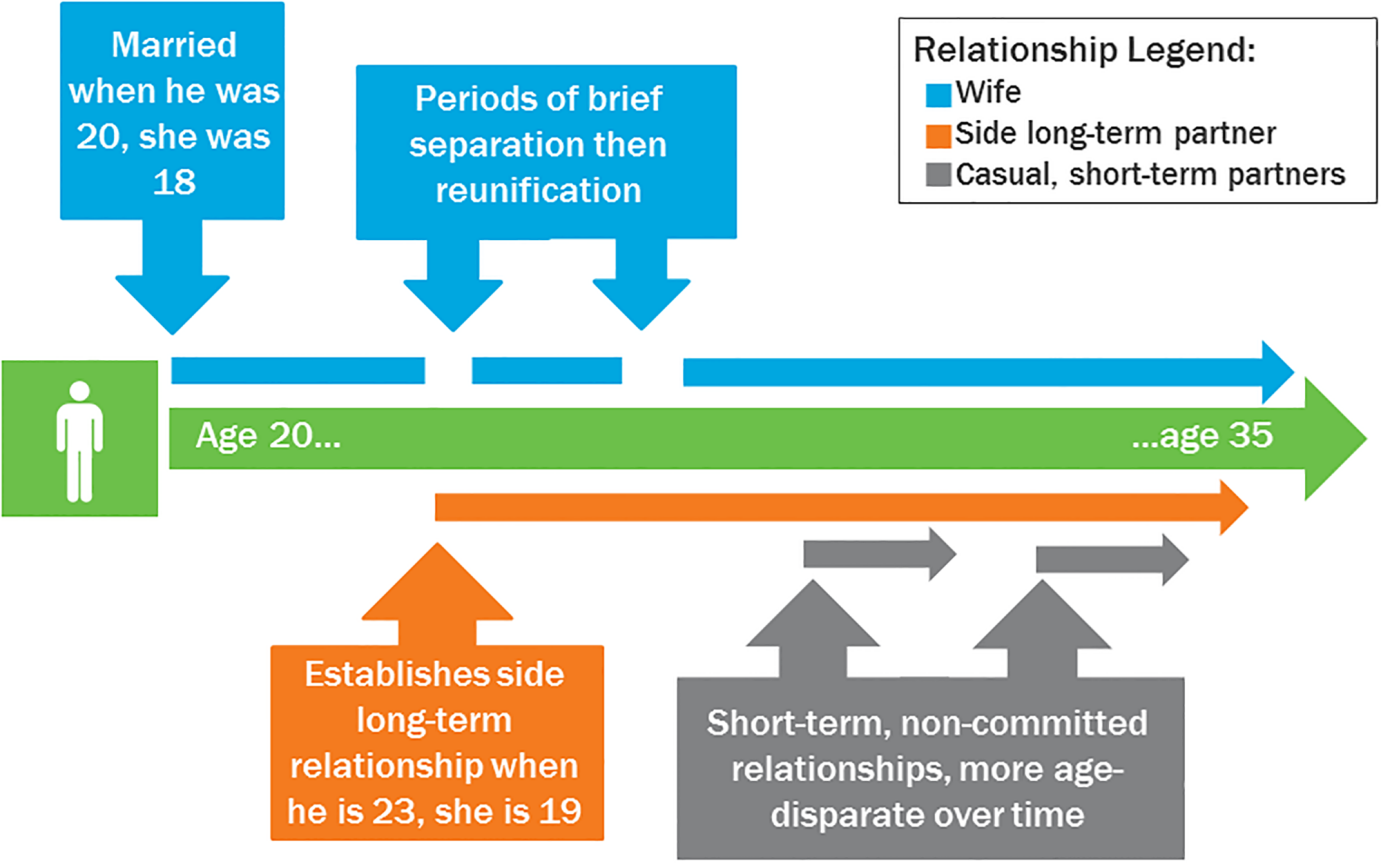
Illustrative summary of men’s complex relationship patterns over time.

Fig 1 summarizes the rather complex patterns of men’s sexual partnerships. The figure shows a typical respondent’s relationships over time from age 20 to age 35, beginning with marrying a first wife, then seeking other long-term and/or short-term partners over time who are even younger than him, while still maintaining his first marriage. The figure is somewhat simplified, since men often noted having multiple long-term side partners, and short-term partners who became long-term partners.

### Men test for HIV at key points in each relationship

Nearly all respondents (95%) reported that they had tested for HIV in the two years prior to the study, and about a quarter of men described frequent testing—that is, testing more than three times in the past two years. Only five men described resistance to testing or hesitancy to test. We did not find major differences in patterns of testing between the three districts. In addition, testing was ubiquitous both among men recruited via their AGYW partners in DREAMS, and men recruited at PTA venues in the communities.

Men’s decisions about HIV testing mapped closely onto key moments in each of their relationships, and couples testing was described about twice as frequently as testing individually.

“…my wife and I have tested together 4 times. The first time was when we had just made the decision to start living together, so we wanted to be sure. The second time was when she said she doesn’t trust my work place so she needs to double check my status. The third time was during ANC [antenatal care] and the fourth time was when there was an outreach near our home.”

(Age 40, Mukono)

Respondents described five key points for HIV testing within the context of a relationship; we describe these below. While not all men described testing at each of these points, most described a number of them for any one partner.

#### Testing before having sex for first time

A number of participants described testing with a new partner before they had sex for the first time. These were mainly married men who were checking on a new side partner, not wanting to bring HIV into their marital relationship.

“We had not even had sex yet…We entered and I just called out the doctor and told him to help us by checking out blood…I don’t want to take the virus to my wife who is at home.”

(Age 22, Gulu)

#### Testing when wanting to stop using condoms

Many men described using condoms early in a relationship if they were unsure of each other’s HIV status and as a family planning method. However, if they realized they may want to stay in the relationship longer-term, they described testing for the explicit purpose of being able to stop using condoms.

“We used to use condoms but that was for the time we didn’t have all the trust for each other and we had not yet tested our blood, but after that we now resorted to other methods that would not make us get unwanted pregnancies.”

(Age 25, Gulu)

#### Testing to solidify relationship, decide to live together, and/ or to enter to marriage

Men described getting to a phase in a relationship where he or the couple realized that they wanted to stay in the relationship longer-term, and wanted to test to know their HIV status first. Often this was also described as way of gauging the character/trustworthiness of the other person (on both sides). One man recounted that *“…after choosing to stay in a relationship*, *it was necessary that we ascertain our sero status so we went for an HIV test*.*” (Age 31*, *Sembabule)* In addition, testing was described as a prerequisite for getting married: *“…I wanted to marry my wife so we had to test*.*” (Age 27*, *Mukono)*

#### Testing due to distrust or after a separation

A number of men described a longer-term partner expressing distrust of his behavior due to recent travel or the nature of his workplace, and therefore suggesting testing. At times this included when the partners had been separated. Sometimes this also involved distrust of or uncertainty about the woman’s status, or mutual distrust of each other’s status. Interestingly, these situations were not described as anxiety-producing, but rather more ‘par for the course’ since it was generally seen as impossible to be sure of a partner’s faithfulness.

“[My wife] did not trust me because people know that we fishermen have several relationships and so it was difficult to convince her that I am not infected.”

(Age 26, Mukono)

“The two times I have chosen to do the test I go with her since she spends a long time without visiting me and I might not know her status.”

(Age 22, Sembabule)

#### Testing during antenatal care

About half the respondents described having tested as part of antenatal care with their partners. These men mainly described testing with their wives/partners when they both went to an antenatal appointment; fewer described their partner going to get them to bring them to antenatal testing.

“The government announced that when a woman becomes pregnant, the man should go with his wife and they are both tested for HIV. So that is what we did when she became pregnant.”

(Age 32, Mukono)

**Fig 2:**
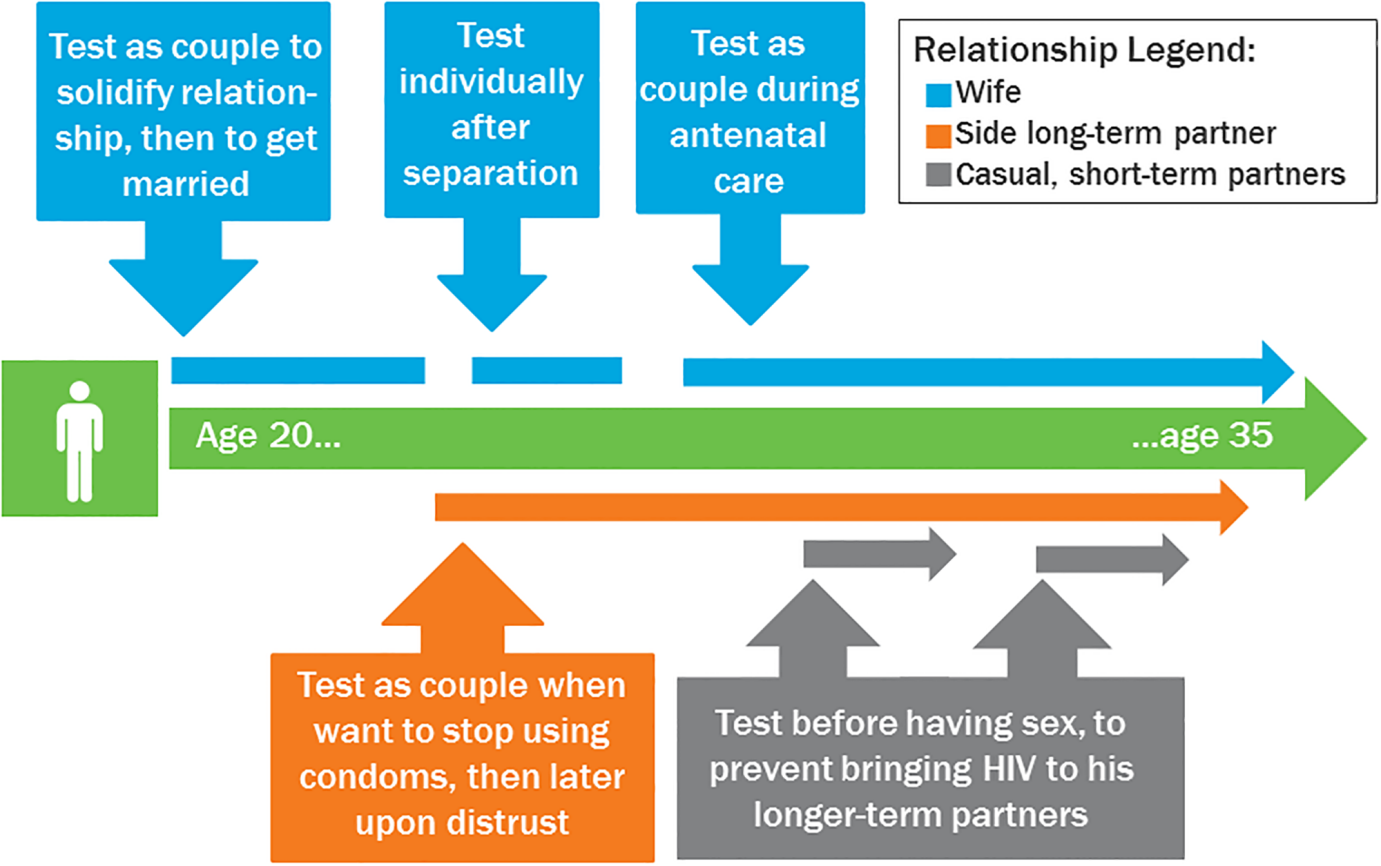
Illustrative case study of a man’s testing over time (using same example as in Fig 1).

Fig 2 expands on the relationship patterns presented in Fig 1 and maps onto the man’s descriptions of HIV testing patterns within their relationships over time. As the number of wives, side, or short-term partners grows, so do the opportunities for risk.

Overall, most men we interviewed were trying to be vigilant about HIV testing and strategic about when they test. However—with the possible exceptions of testing before first having sex, and testing to stop using condoms—the motivation behind this vigilance seemed more due to trying to establish or maintain trust in the relationship rather than preventing HIV.

“I did not trust her as we had not had an HIV test together. You may also find that she did not trust me because she did not know much about me. So everybody was suspicious of each other…”

(Age 37, Mukono)

Furthermore, for many men, testing seemed to serve as validation for continuing their practice of having multiple concurrent partners; only a few men described the testing experience as prompting them to re-examine their risk behavior. An extreme example of this came from a man who has four wives:

“The fact that I stay away from my wives for a month or more, I don’t sleep with them without taking an HIV test.” [later segment] “My wives fear that I might infect them with the HIV virus, likewise I am also scared of them; you never know what happens while at work and away from them for many days. This arrangement has helped me to prevent HIV infection in my family by conducting an HIV test before I sleep with any of my wives.”

(Age 45, Sembabule)

## Discussion

In this qualitative study with male partners of adolescent girls and young women across three regions of Uganda, we found frequent patterns of multiple concurrent sexual partnerships among the respondents. Just how common and normalized multiple partnerships were in the study settings was further reinforced when it became clear that respondents recruited via their largely marital/cohabiting partners (i.e., AGYW partners enrolled in DREAMS) described multiple relationships as often and as openly as men recruited at PTA venues (where a much smaller proportion reported being married). These findings highlight the practice of multiple concurrent partnerships as a continuing public health concern, reinforcing findings from the 2016 national Demographic Health Survey (DHS) [17] as well as many other recent studies in Uganda [18–23]. Whether and in what ways the pervasiveness of multiple partnerships is a *new* phenomenon, as many respondents framed it, bears further investigation.

We also found substantial variety in the types of relationships described, including formal marital partners, long-term side partners (who were often called ‘wives’), and casual transactional relationships. Furthermore, we found that the nature of relationships was highly fluid—characterized by multiple separations due to conflict or residential location, and reunifications which at each point presented opportunities for finding other new partners. Such relationship fluidity is very difficult to capture in quantitative research, and has not received due attention in HIV prevention literature. Subsequently, public health programs and messaging are often oriented around the notion that multiple partnerships involve only short-term relationships, or a married relationship plus short-term side relationships, which may not be the predominant pattern in Uganda. Public health programs and research in Uganda may need to carefully consider and design programs around the pattern of multiple long-term relationships, as well as the fluidity of these relationships.

Turning to the age of men’s partners, we found that the age gap between men and their first wife (commonly 3–5 years younger) tended to be smaller than the gap with subsequent partners, who continued to be mainly AGYW ages 15–24 even as the men entered their 30s and 40s. Respondents explicitly described a desire to seek out younger women, versus women around their same age. Motivations for this included that younger women could be more easily controlled and that younger women showed men greater care and respect. Such motivations may simultaneously reflect men’s own felt need to maintain control over their female partners, and their sense of uncertainty and vulnerability in relationships [24, 25]. The unequal power dynamics resulting from age- and power-disparate relationships may in turn create high risk for adverse sexual and reproductive health outcomes among AGYW [6, 26, 27]. At times, along with being younger, AGYW partners were experiencing other vulnerabilities that reduced their power in relationships. For example ‘Ogek’, young women from failed marriages, were taken as second or third wives in Gulu, and young sex workers were frequented by men in Sembabule and Mukono. These relationship dynamics point to particular vulnerabilities among these groups of young women which warrant close attention in programs like DREAMS.

Uganda has previous experience with HIV prevention programming targeted toward changing sexual partnership patterns. For instance, Uganda’s promotion of “zero grazing” (an agricultural analogy which promoted staying within one’s existing sexual network) was a key HIV prevention message in the 1990’s and early 2000’s and early declines in HIV rates are in part attributed to this focus [28]. Recent HIV prevention messages in Uganda also address the need to become aware of one’s sexual networks. However, the complexity and fluidity of most men’s relationships—with partners moving in and out of relationships and at the same time starting other new relationships—may render messages focused on staying within one’s existing sexual network ineffective in preventing HIV transmission. It may be that a stronger emphasis on improving interpersonal communication and trust in relationships is warranted [20]. A potential intervention model in Uganda is the SASA! intervention which was first implemented and rigorously evaluated in Kampala, and has since been adopted in many countries in the region. SASA! is a community mobilization effort aimed at preventing intimate partner violence and reducing HIV risk behaviors, which was evaluated in a cluster randomized controlled trial from 2007–12. It resulted in a substantial reduction in sexual partner concurrency among men, largely by improving levels of communication, trust and intimacy within relationships [29]. Another promising intervention in Uganda, the Parenting for Respectability Programme, which has been well received by men, focuses on building positive spousal relationships within a positive parenting and gender based violence prevention program [30].

HIV testing was very commonly reported among our respondents, regardless of geographic district or recruitment strategy, which was a surprise to the study team. Despite high numbers of sexual partnerships and rates of partner concurrency in Uganda, previous study findings have documented men’s relatively low testing rates and strong reluctance to test [3, 14, 17, 31]. Further, men in our study described remarkable vigilance about testing with each partner—usually not just once but at a number of key points of the relationship—to check or re-check that he was HIV-negative, and to confirm a new partner’s status before initiating unprotected sex with her. Such an orientation towards testing has long been promoted in HIV prevention programs and should continue to be promoted.

Nonetheless, our findings also suggest that the complexity and fluidity of relationships in Uganda may be complicating individuals’ and couples’ preventive decision-making. As an example, a man may test with a recent side partner in order to stop using condoms with her, but he may not have recently tested with his wife or other partners and likely isn’t consistently using condoms with them, creating openings for HIV transmission. Additionally, in most cases, the ways in which men described testing suggested that it served to validate their continuation of current sexual relationship practices rather than prompting a reconsideration of these practices. This finding is in line with other recent research in Uganda suggesting that while women adhered to public health messages promoting faithfulness as a means of HIV risk reduction, this was not the case with men [32]. Rather than only sending clients who test negative on their way with a recommendation to retest and use condoms, testing and counseling strategies should support men to reduce risk and build healthier relationships [33]. Indeed, testing could serve as an important entry point into other relationship strengthening strategies.

An additional aim of this study was to look at differences in relationships and HIV service use across Gulu, Mukono and Sembabule—districts in regions of the country with considerable cultural and economic diversity. With the exceptions noted above, overall we found that patterns and dynamics of relationships were remarkably similar across these three districts. The same was true for patterns of HIV testing, including frequency of testing, couples testing, and points in relationships when testing occurred. These findings suggest that similar interventions could be effective across the three regions, and that there may be less need for tailoring strategies and messaging to particular localities than was originally expected.

This manuscript has several limitations worth noting. First, participants’ responses may reflect social desirability bias, as do any self-report data. Second, we are unable to provide an exact estimate of prevalence of multiple sexual partners, as opposed to multiple concurrent partners. Most men described their recent partnerships in ways that suggested their partnerships were overlapping in time (i.e., concurrent); however we cannot discern with certainty, based on men's qualitative descriptions, how many relationships were not in fact overlapping (i.e., separate out multiple partners from multiple concurrent). Third, while we focus on overarching themes of multiple partnerships and HIV testing in this manuscript, there are a number of additional themes that could be further explored in this expansive qualitative data (for instance, around relationship motivations and conflict). We plan to pursue some of these topics in subsequent analyses and manuscripts. Finally, despite the large sample size for a qualitative study and coverage of three diverse districts, the sample may not be representative of adult men or male partners of AGYW in the three districts or across Uganda. Key strengths of our study are that findings are from men’s perspectives and capture rich descriptions of their experiences over time, through a large number of qualitative interviews across diverse regions of the country.

## Conclusions

Our findings highlight the centrality of concurrent, fluid partnerships in shaping HIV risk among men and their young female partners across the three diverse study districts in Uganda. Re-emphasizing primary prevention, and particularly improving communication and trust in relationships, alongside biomedical prevention approaches appears critical for limiting new HIV infections in Uganda [2]. In addition, while men’s vigilance around and frequency of HIV testing is undoubtedly a positive sign, it is clear that for most men these testing experiences are not serving as an entry point to risk reduction. Testing and counseling strategies should support men to reduce risk and build healthier relationships—returning HIV counseling and testing to its intended purpose as an essential gateway to both HIV treatment and prevention.

## References

[1] UNAIDS. The Gap Report. Geneva: UNAIDS, 2014.

[2] Isbell MT, Kilonzo N, Mugurungi O, Bekker L-G. We neglect primary HIV prevention at our peril. The Lancet HIV. 2016;3(7):e284–e5. 10.1016/S2352-3018(16)30058-3 27365201

[3] Ugandan AIDS Commission. The HIV and AIDS Uganda Country Progress Report 2014. 2015.

[4] PEPFAR. PEPFAR 2015 Annual Report to Congress. 2015.

[5] Francisco L, Abramsky T, Kiss L, Michau L, Musuya T, Kerrigan D, et al Violence Against Women and HIV Risk Behaviors in Kampala, Uganda Baseline Findings from the SASA! Study. Violence against women. 2013;19(7):814–32. 10.1177/1077801213497557 23955928

[6] Leclerc-Madlala S. Age-disparate and intergenerational sex in southern Africa: the dynamics of hypervulnerability. Aids. 2008;22:S17–S25. 10.1097/01.aids.0000341774.86500.53 19033752

[7] Pulerwitz J, Michaelis A, Verma R, Weiss E. Addressing gender dynamics and engaging men in HIV programs: lessons learned from Horizons research. Public health reports. 2010:282–92. 10.1177/003335491012500219 20297757 PMC2821858

[8] de Oliveira T, Kharsany AB, Gräf T, Cawood C, Khanyile D, Grobler A, et al Transmission networks and risk of HIV infection in KwaZulu-Natal, South Africa: a community-wide phylogenetic study. The Lancet HIV. 2017;4(1):e41–e50. 10.1016/S2352-3018(16)30186-2 27914874 PMC5479933

[9] Higgins JA, Hoffman S, Dworkin SL. Rethinking gender, heterosexual men, and women's vulnerability to HIV/AIDS. American journal of public health. 2010;100(3):435–45. 10.2105/AJPH.2009.159723 20075321 PMC2820057

[10] UNAIDS. Get on the fast track: the life-cycle approach to HIV. Geneva, Switzerland. 2016.

[11] PEPFAR. Working together for an AIDS-Free Future for Girls and Women: The DREAMS Partnership 2018 [cited 2018 February 11]. https://www.pepfar.gov/partnerships/ppp/dreams/.

[12] Cornell M, McIntyre J, Myer L. Men and antiretroviral therapy in Africa: our blind spot. Tropical Medicine & International Health. 2011;16(7):828–9. 21418449 10.1111/j.1365-3156.2011.02767.x PMC3749374

[13] Mills EJ, Ford N, Mugyenyi P. Expanding HIV care in Africa: making men matter. The Lancet. 2009;374(9686):275–6. 10.1016/S0140-6736(09)61348-9 19632481

[14] Siu GE, Seeley J, Wight D. Dividuality, masculine respectability and reputation: how masculinity affects men's uptake of HIV treatment in rural eastern Uganda. Social Science & Medicine. 2013;89:45–52. 23726215 10.1016/j.socscimed.2013.04.025

[15] Patton MQ. Qualitative research & evaluation methods: Integrating theory and practice. Sage, Thousand Oaks, CA 2015.

[16] Creswell JW, Poth CN. Qualitative inquiry and research design: Choosing among five approaches: Sage publications; 2017.

[17] Uganda Bureau of Statistics & The DHS Program ICF. Uganda Demographic and Health Survey 2016. Kampala, Uganda & Rockville, Maryland USA: 2017.

[18] Seeley J, Nakiyingi-Miiro J, Kamali A, Mpendo J, Asiki G, Abaasa A, et al High HIV incidence and socio-behavioral risk patterns in fishing communities on the shores of Lake Victoria, Uganda. Sexually transmitted diseases. 2012;39(6):433–9. 10.1097/OLQ.0b013e318251555d 22592828

[19] Rutakumwa R, Mbonye M, Kiwanuka T, Bagiire D, Seeley J. Why do men often not use condoms in their relationships with casual sexual partners in Uganda? Culture, health & sexuality. 2015;17(10):1237–50. 10.1080/13691058.2015.1053413 26158527

[20] Higgins JA, Mathur S, Eckel E, Kelly L, Nakyanjo N, Sekamwa R, et al Importance of relationship context in HIV transmission: results from a qualitative case-control study in Rakai, Uganda. American journal of public health. 2014;104(4):612–20. 10.2105/AJPH.2013.301670 24524490 PMC4025686

[21] Grabowski MK, Serwadda DM, Gray RH, Nakigozi G, Kigozi G, Kagaayi J, et al HIV Prevention Efforts and Incidence of HIV in Uganda. New England Journal of Medicine. 2017;377(22):2154–66. 10.1056/NEJMoa1702150 29171817 PMC5627523

[22] Matovu J, Ssebadduka B. Sexual risk behaviours, condom use and sexually transmitted infection treatment-seeking behaviours among female sex workers and truck drivers in Uganda. International journal of STD & AIDS. 2012;23(4):267–73. 22581951 10.1258/ijsa.2011.011313

[23] Santelli JS, Edelstein ZR, Mathur S, Wei Y, Zhang W, Orr MG, et al Behavioral, biological, and demographic risk and protective factors for new HIV infections among youth, Rakai, Uganda. Journal of acquired immune deficiency syndromes (1999). 2013;63(3):393. 23535293 10.1097/QAI.0b013e3182926795 PMC4131841

[24] Gottert A, Barrington C, McNaughton-Reyes HL, Maman S, MacPhail C, Lippman SA, et al Gender Norms, Gender Role Conflict/Stress and HIV Risk Behaviors Among Men in Mpumalanga, South Africa. AIDS and Behavior. 2017:1–12. 10.1007/s10461-017-1706-9 PMC6440537 28161801

[25] Mathur S, Higgins JA, Thummalachetty N, Rasmussen M, Kelley L, Nakyanjo N, et al Fatherhood, marriage and HIV risk among young men in rural Uganda. Culture, health & sexuality. 2016;18(5):538–52. 10.1080/13691058.2015.1091508 PMC4897968 26540470

[26] Longfield K, Glick A, Waithaka M, Berman J. Relationships between older men and younger women: implications for STIs/HIV in Kenya. Studies in family planning. 2004;35(2):125–34. 15260214 10.1111/j.1728-4465.2004.00014.x

[27] Ritchwood TD, Hughes JP, Jennings L, MacPhail C, Williamson B, Selin A, et al Characteristics of Age-Discordant Partnerships Associated With HIV Risk Among Young South African Women (HPTN 068). Journal of acquired immune deficiency syndromes. 2016;72(4):423–9. 10.1097/QAI.0000000000000988 26977748 PMC4925181

[28] Green EC, Halperin DT, Nantulya V, Hogle JA. Uganda's HIV prevention success: the role of sexual behavior change and the national response. AIDS and Behavior. 2006;10(4):335–46. 10.1007/s10461-006-9073-y 16688475 PMC1544373

[29] Abramsky T, Devries K, Kiss L, Nakuti J, Kyegombe N, Starmann E, et al Findings from the SASA! Study: a cluster randomized controlled trial to assess the impact of a community mobilization intervention to prevent violence against women and reduce HIV risk in Kampala, Uganda. BMC medicine. 2014;12(1):122. 25248996 10.1186/s12916-014-0122-5 PMC4243194

[30] Siu GE, Wight D, Seeley J, Namutebi C, Sekiwunga R, Zalwango F, et al Men’s Involvement in a Parenting Programme to Reduce Child Maltreatment and Gender-Based Violence: Formative Evaluation in Uganda. The European Journal of Development Research. 2017;29(5):1017–37.

[31] Siu GE, Wight D, Seeley JA. Masculinity, social context and HIV testing: an ethnographic study of men in Busia district, rural eastern Uganda. BMC Public Health. 2014;14(1):33. 24417763 10.1186/1471-2458-14-33 PMC3893584

[32] Mathur S, Romo D, Rasmussen M, Nakyanjo N, Nalugoda F, Santelli JS. Re-focusing HIV prevention messages: a qualitative study in rural Uganda. AIDS research and therapy. 2016;13(1):37. 27857775 10.1186/s12981-016-0123-x PMC5105323

[33] World Health Organization. Consolidated strategic information guidelines for HIV in the health sector. Geneva: 2015. 26110192

